# Bronchiectasis in PCD looks different to CF on CT scan

**DOI:** 10.1186/s40248-018-0139-2

**Published:** 2018-08-09

**Authors:** Philip Robinson, Lucy Morgan

**Affiliations:** 1 Department of Respiratory and Sleep Medicine, Royal Children’s Hospital, Parkville, Melbourne, 3052 Australia; 2 Department of Paediatrics, University of Melbourne, Melbourne, Australia; 3 Murdoch Children’s Research Institute, Melbourne, Australia; 4 Department of Respiratory Medicine, Concord Hospital, Sydney, Australia; 5 School of Medicine, University of Sydney, Sydney, Australia

**Keywords:** Cystic fibrosis, Primary ciliary dyskinesia, CT scanning, Bronchiectasis

## Abstract

The contemporary diagnosis of bronchiectasis requires CT scanning to describe specific structural lung changes. Scoring systems have been designed and validated in some specific causes of bronchiectasis to allow investigators to correlate CT changes with other indices of disease severity, to describe changes over time, with exacerbation and in response to treatment. Several scoring systems have been developed for CF including the Bhalla Score, Brody score, and the Helbich score. These scoring systems have also been applied to other causes of bronchiectasis including Primary Ciliary Dyskinesia (PCD). This assumes that the nature of structural lung disease in these conditions, as well as the rate and nature of longitudinal changes are identical to, or at least very similar to, those found in CF. This assumption has not been tested. The underlying pathophysiology of PCD is not the same as CF and may cause bronchiectasis that is radiologically similar but not necessarily the same as CF or any other cause of bronchiectasis.

The development of a disease specific scoring system for structural change in cases of non-CF bronchiectasis due to any cause, would require consideration of the full range of changes seen in that condition without reference to changes seen in other conditions. We present a summary of structural findings that have been described in PCD and highlight the radiological differences between PCD and other causes of bronchiectasis. We suggest that a PCD specific CT scoring system is required to properly describe changes seen in PCD.

## Structural lung disease in PCD – is the use of CF derived scoring systems appropriate?

The structural changes seen on pulmonary CT scans from patients with Cystic Fibrosis (CF) and Primary Ciliary Dyskinesia (PCD), both causes of significant bronchiectasis, have been the basis of numerous previous reports [1–6]. More information on the range of these structural changes is available from patients with CF, due in part to the ability to detect CF by new born screening and because early treatment of this common genetic disease has the potential to prevent or retard the onset of bronchiectasis. In contrast, PCD (which is much less common) has no new-born screen available. The diagnosis is often made much later by which time symptoms are more significant and structural lung damage has often already developed [7].

CT scoring systems have been developed and validated to facilitate clinical research and improve clinical care. All the current scoring systems were developed by describing the range and frequency of radiological abnormalities and a ranked hierarchy of these features. Almost exclusively the scores established for suppurative lung disease, particularly in the paediatric setting, have been established using scans performed on patients with CF. These scoring systems have been clearly and credibly validated and have played an important role in the evolution of best practice clinical guidelines for CF. However, the utility of CF CT scoring systems for non CF bronchiectasis is less clear.

PCD is an important cause of bronchiectasis and there are several reports that describe the radiological features of PCD-bronchiectasis [2–6, 8]. Most groups have used CF derived, scoring systems to describe these changes. It is clear that PCD and CF lead to bronchiectasis via distinctly different pathophysiology. It is not as clear that the two diseases lead to the same structural lung damage in the same time frame. It is plausible that utilising these CF- derived scoring systems will provide an incomplete picture of this specific cause of bronchiectasis.

Neonatal respiratory distress in a term infant without evidence of sepsis is strongly suggestive of PCD and is found in upwards of 65–70% of infants with PCD [9]. First reported as an association by Nichamin in 1956, Mullowney has more recently described the radiological findings of 23 infants with PCD who had a chest X-ray in the face of neonatal respiratory difficulty [10, 11]. Seventy percent of the infants showed evidence of lobar collapse – 75% of which occurred in the upper lobes. The preponderance of upper lobe disease in PCD in early life is in contrast to changes seen in infants with Cystic Fibrosis. Sly and colleagues in a study of 57 infants with CF detected by new born screening reported that 80% of CT scans performed soon after diagnosis showed evidence of structural lung disease [1]. While bronchial dilatation was seen in 18.6% of scans and bronchial wall thickening in 45%, lobar collapse was not reported. In addition the changes seen were not present in any greater incidence in upper lobes compared to lower lobes suggesting that upper lobe predominance is an important discriminator between CF and PCD in this age group.

Over the past decade several groups have described structural changes seen on CT scans from older patients with PCD. All reports have used CF derived scoring systems to describe such changes. The three most commonly used scoring systems are the Bhalla, Brody (and modified Brody) and Heilbeich scoring systems. A brief analysis of how these scores were developed is relevant when considering their suitability for use in non CF causes of bronchiectasis such as PCD [12–14].

The Bhalla score, reported in 1991, was derived from consideration of CT scans from 14 patients with CF ranging from 5 to 42 years old [12]. In contrast the original Brody score was developed in 1999 and subsequently modified for use in an imaging sub-study associated with the Pulmozyme Early Intervention Trial (PEIT) in 2004 [13, 14]. Patients received CT scans before, and after 2 years of Pulmozyme inhalation. The study involved 60 children between the ages of 6 and 10 with well-preserved lung function at enrolment. Subsequent modifications of the Brody score have been developed and used in a much wider range of patients with CF. The Heilbeich scoring system, reported in 1999, was developed using pulmonary CT findings from 117 patients with CF. Cases were classified according to age; 0–5 years, 6–16 years, and 17 years and older and the frequency of a prespecified list of CT abnormalities were recorded [15]. The severity and anatomic extent of each of these features were used to generate an overall score.

In 2007, Jain et al. reported the spectrum of structural changes seen on CT scans from 26 children with PCD using a modified Brody score [3]. In addition to the previously reported changes seen in CF including bronchiectasis and bronchial wall thickening, the authors described the relative sparing of upper lobes from disease, particularly bronchiectasis. Overall severity scores were highest for the middle and lingula lobes. It is notable that the relative sparing of the upper lobes is not a part of any CF derived scoring system. Santamaria et al. in 2008 also used a modified Brody score to evaluate structural lung disease in 20 children and adults with PCD [4]. Bronchiectasis was seen in 80% of cases while peribronchial thickening (80%) mucus plugging (75%); parenchymal changes (65%) were also seen commonly. In comparison to a group of age matched CF patients, the total HRCT scan score in PCD was significantly lower.

In 2012 Magnin et al. examined structural changes on CT scans in 20 adolescents with PCD using a modified Brody and Bhalla scoring system [5]. Bronchiectasis was present in 70% of scans, and peribronchial thickening in 90% of scans. CT scores significantly worsened with age, and were negatively correlated to measurements of lung function. Similar findings were described by Cohen-Cymberknoh et al. in 2014 when lung function and CT changes were assessed, using the Brody score, in children with PCD and 2 groups of CF patients [2]. More recently Maglione reported on findings on both CT scan and MRI scanning in a group of 20 young adolescents with PCD and 20 age similar patients with Cystic Fibrosis using the Helibich scoring system [6].

All the studies summarised in this review describing structural lung disease in PCD have assumed, through the use of CF derived scoring systems, that the range of changes present in PCD are identical to those found in Cystic Fibrosis. The possibility that unique structural changes may occur in PCD has not been considered through the use of these CF derived scores.

Recently we reviewed 19 volumetric CT scans performed on adolescents and young children (mean age 11.7 ± 3.3 years, 9 males) with PCD. All patients had a definitive diagnosis of PCD according to diagnostic criteria recently described [16]. Patients over 5 had at least 2 of the major clinical symptoms of PCD: unexplained neonatal respiratory distress, evidence of chronic upper or lower airway suppurative disease – productive cough or recurrent sinusitis/coryza or laterality defects. Patients also had at least 2: low nasal NO, presence of bi-allelic PCD-causing genes, ultrastructural ciliary abnormalities on electron microscopy, or ciliary waveform abnormalities recognised in PCD seen on high speed video-microscopy. Younger patients had similar requirements but without nasal NO measurement. Scans were scored by an experienced paediatric respiratory physician (P.R.) with experience in CT scoring [17–19]. No attempt was made to score the scans using CF derived scoring systems. Instead, we started from first principles and described the range and frequency of radiological abnormalities present and a ranked hierarchy of these features. The previously reported sparing of bronchiectasis in upper lobes was again evident with only 3 upper lobes of 38 (8%) showing bronchiectasis compared to 35 of 38 lower lobes (92%). Middle lobe bronchiectasis was present in 30 of the 38 lobes (79%). Several unique features were evident in the PCD scans which are not commonly seen in CF. Linear atelectasis was seen in 27 of the 162 lobes (16%) with findings almost exclusively limited to the middle lobes (75%). No linear atelectasis was seen in upper lobes. Interlobar septal thickening was seen in 33% of scans. These findings are not commonly seen in scans performed on patients with CF and, where present, not in the similar frequency or with upper lobe sparing seen in PCD [14].

## Conclusion

This preliminary data suggests that structural lung disease in PCD has unique features both in lobar localisation and structural nature and that the use of CF derived scoring systems which do not take these features into consideration may potentially result in incomplete assessment of the bronchiectasis associated with PCD. In order to accurately describe the range of structural changes seen on CT scans in PCD, and indeed the evolution of these changes over time, a scoring system based solely on findings from PCD CT scans should be developed using the same multicentre, international, large scale strategy used to develop the CF CT scores.

While future work may see the replacement of CT scanning by different MRI scanning modalities, including the use of hyper polarised gases such as Hyper-polarised Helium or Xenon, the assessment of observed structural changes, regardless of the screening modality should be based on PCD derived systems.
